# Aims and structure of the German Research Consortium BipoLife for the study of bipolar disorder

**DOI:** 10.1186/s40345-016-0066-0

**Published:** 2016-11-21

**Authors:** Philipp S. Ritter, Felix Bermpohl, Oliver Gruber, Martin Hautzinger, Andreas Jansen, Georg Juckel, Tilo Kircher, Martin Lambert, Christoph Mulert, Andrea Pfennig, Andreas Reif, Otto Rienhoff, Thomas G. Schulze, Emanuel Severus, Thomas Stamm, Michael Bauer

**Affiliations:** 1 Department of Psychiatry and Psychotherapy, University Hospital Carl Gustav Carus, Technische Universität Dresden, Fetscherstr. 74, 01307 Dresden, Germany; 2 Department of Psychiatry, Charité im St. Hedwig-Krankenhaus, Große Hamburger Str. 5, 10115 Berlin, Germany; 3 Department of Psychiatry, Ruprecht-Karls-University Heidelberg, Voßstr. 4, 69115 Heidelberg, Germany; 4 Department of Psychology Clinical Psychology and Psychotherapy, Eberhard Karls University, Schleichstr. 4, 72076 Tübingen, Germany; 5 Department of Psychiatry and Psychotherapy, Philipps-University Marburg, Marburg, Germany; 6 Marburg & Core-Unit Brain Imaging, Philipps-University Marburg, Rudolf-Bultmann-Str. 8, 35039 Marburg, Germany; 7 Department of Psychiatry, Psychotherapy and Preventive Medicine, LWL University Hospital, Ruhr University Bochum, Bochum, Germany; 8 Department of Psychiatry and Psychotherapy, Centre of Psychosocial Medicine, Psychosis Centre, University Medical Center Hamburg-Eppendorf, Hamburg, Germany; 9 Department of Psychiatry, Psychosomatic Medicine and Psychotherapy, University Hospital Frankfurt, Frankfurt, Germany; 10 Department of Medical Informatics, University of Göttingen, Göttingen, Germany; 11 Institute of Psychiatric Phenomics and Genomics (IPPG), Medical Center of the University of Munich, Munich, Germany

**Keywords:** Bipolar disorder, Randomized controlled trial, Research consortium, Psychotherapy, Early recognition, Ambulatory monitoring, BipoLife

## Abstract

**Background:**

Bipolar disorder is a severe and heterogeneous mental disorder. Despite great advances in neuroscience over the past decades, the precise causative mechanisms at the transmitter, cellular or network level have so far not been unraveled. As a result, individual treatment decisions cannot be tailor-made and the uncertain prognosis is based on clinical characteristics alone. Although a subpopulation of patients have an excellent response to pharmacological monotherapy, other subpopulations have been less well served by the medical system and therefore require more focused attention. In particular individuals at high risk of bipolar disorder, young patients in the early stages of bipolar disorder, patients with an unstable highly relapsing course and patients with acute suicidal ideation have been identified as those in need.

**Structure:**

A research consortium of ten universities across Germany has therefore implemented a 4 year research agenda including three randomized controlled trials, one epidemiological trial and one cross-sectional trial to address these areas of unmet needs. The topics under investigation will be the improvement of early recognition, specific psychotherapy, and smartphones as an aid for early episode detection and biomarkers of lithium response. A subset of patients will be investigated utilizing neuroimaging (fMRI), neurophysiology (EEG), and biomaterials (genomics, transcriptomics).

**Conclusions:**

This article aims to outline the rationale, design, and methods of these individual studies.

## Background

Bipolar disorder is a severe and recurrent mental disorder ranked among the leading causes of disability. The condition has a high degree of heritability (Kieseppa et al. 2004) and is associated with poor social and occupational outcomes, high rates of morbidity and mortality and is considered to be one of the disorders with the highest risk of suicide (Chen and Dilsaver 1996). Although there have been great advances in the understanding of mental disorders in general, the precise mechanisms underlying bipolar disorder and the response to treatment at the genetic, cellular or neuroanatomical level remain elusive.

The heterogeneity of the disorder and the complexity of its neurobiological underpinnings require an integrated multicentre approach to research, utilizing diverse research strategies and harvesting the access to substantial patient numbers that could not be recruited at a single site alone.

The consortium of ten university hospitals described in this article has defined areas of unmet needs in prevention, diagnosis and therapeutic intervention for four subgroups of patients: individuals at high risk of bipolar disorder, young patients in the early stages of bipolar disorder, patients with an unstable highly relapsing course, and patients with acute suicidal ideation.

While a subgroup of patients with bipolar disorder responds excellently to lithium monotherapy, treatment response remains inadequate for most and no biomarkers exist to guide a rational choice of treatment (Kessing et al. 2011). A fifth project within the consortium will therefore attempt to identify genomic, transcriptomic, and proteomic markers of lithium response.

Three randomized controlled trials (RCTs), one epidemiological trial, and one cross-sectional trial investigating biomaterials will be supported by two translational platforms supplying the infrastructure for multicentre data-management, bio banking, electroencephalography, and neuroimaging. The consortium incorporates nine German university hospitals (Dresden, Berlin, Munich, Frankfurt, Marburg, Hamburg, Bochum, Tubingen, and Heidelberg) and the medical informatics section of Gottingen University.

The networks 4 year funding period by the German ministry of education (BMBF) began in 2015.

Each project will be introduced by outlining the rationale, the questions the study will attempt to clarify, and a synopsis of the study protocol.

### Improving early recognition and intervention in people at-risk of developing bipolar disorder (Project A1)

A large proportion of patients with bipolar disorder (BD) experience substantial symptomatology months or even years before full BD manifestation (Lish et al. 1994). Studies have repeatedly demonstrated a significant delay until adequate diagnosis and treatment, estimated to be app. 10 years on average (Baldessarini et al. 2003; Pfennig et al. 2011). Treatment delay is associated with a poor functional outcome and an elevated risk of suicide (Post et al. 2010). Delaying the initiation of lithium is equally associated with a poorer response (Kessing et al. 2014). Prolonged periods of undetected illness with no or inadequate treatment leading to significant psychosocial impairment therefore necessitate improved instruments and guidelines for early identification and intervention so as to advance overall disease management.

So far there are no evidence-based guidelines for primary or secondary prevention of BD. Only a few small interventional studies investigating pharmacological interventions (placebo-controlled trials: lithium (Geller et al. 1998), valproic acid (Findling et al. 2007) or family focused psychotherapy (Miklowitz et al. 2011; Miklowitz et al. 2013; Nadkarni and Fristad 2010) have been conducted, with results that are insufficiently reliable because of several limitations, particularly insufficient power, and the lack of an adequate control condition within the psychotherapy trials.

Due to its high heritability a positive family history remains as one of the major risk factors for BD. In offspring of parents with BD, there is evidence that anxiety is an antecedent of subsequent mood episodes (Duffy et al. 2007). Furthermore, clinical, epidemiological, and genetic data suggest that the risk to develop BD is increased in at least a subgroup of patients with ADHD (Duffy 2012), and a diagnosis of substance abuse, insomnia (Ritter et al. 2015) and a history of stressful life events may be precursors of subsequent BD. Among patients with single or recurrent depression, certain psychopathological markers (i.e., suicidality and diurnal variation) are associated with a higher incidence of ensuing manic episodes (Pfennig et al. 2016).

Early recognition and risk stratification efforts began in the domain of psychotic disorders and have only recently been expanded to BD. Preliminary structured instruments have been developed to map potential risk factors, quantify the risk, and evaluate the predictive power for conversion prospectively (BPSS-P, EPIbipolar, BAR criteria) (Correll et al. 2014; Leopold et al. 2012; Bechdolf et al. 2012) and attempts are being made to identify reliable biomarkers (Duffy et al. 2014). The available limited prospective data in small samples need to be substantiated by a longitudinal approach in larger at-risk cohorts. Furthermore, resilience factors (i.e., emotion regulation processes) have received very little attention so far.

Project A1 therefore aims to address the following questions:

- What is the predictive power of individual risk factors and risk constellations in defined risk groups for BD in the age group 15–35 and in a representative cohort using the different existing instruments/criteria?
- What are resilience factors that lower the risk for BD in the proposed age group?
- What is the association of fluid/non-fluid biomarkers and neuroimaging data with clinical outcome?
- How can the information from (a), (b), and (c) be integrated for further development of the diagnostic tools and harmonization of the diagnostic process across centers?
- What factors describe and/or influence the process of treatment decision-making in this naturalistic setting? What are the efficacy (acute/preventive effects) and tolerability/safety measures of treatment approaches in at-risk subjects?
- What information from (e) can be used to refine a proposed category model & treatment guidance?

We are conducting a multicentre, prospective, naturalistic cohort study with a follow-up of ≥24 months per individual. Three groups are being studied: (a) Help-seeking youth and young adults aged 15–35 without a diagnosis of BD consulting early detection centers & specialized services with ≥1 proposed risk factor for BD; (b) In-/outpatients with a depressive episode aged 15–35; (c) In-/outpatients with ADHD aged 15–35.

As reference group for the frequency of the potential risk factors, the German IMAGEN cohort, a representative population cohort, is being assessed. The cohort was first assessed at age 14 and 16 and is currently being re-assessed at age 18–20.

All participants will receive state of the art counseling and treatment according to their individual needs. This will encompass general preventive measures, specific preventive measures, psychotherapy, and/or pharmacotherapy. The assessments utilized will include a detailed description of current and socioeconomic circumstances, the above-mentioned instruments to assess risks of subsequent BD and instruments to assess resilience, psychosocial functioning, and comorbidities. The baseline assessment will be followed by biannual follow-ups for a minimum of 2 years. Participants will receive the option of participating in the imaging, EEG, and genomics studies (technical platform projects, TPP1 and TPP2) with the aim of identifying potential neuroimaging, neurophysiological, and genomic markers for the prediction of subsequent conversion to bipolar disorder. The details of the methods and paradigms used are described in section TPP1 and TPP2.

The statistical assumptions for the two main hypotheses are as follows: *Hypothesis 1* Over a period of ≥24 months, ≥15% of at-risk individuals will develop a first (hypo)manic episode. The assumption of a 15% conversion rate is generally considered conservative. *Hypothesis 2* Specific risk factors or risk factor constellations will be predictive of mania development during the observation period. We will test this hypothesis in various ways. First, univariate analyses will be run to determine individual predictors of conversion to BP-I. Second, we will conduct a multivariate logistic regression model to determine risk factor constellations that are predictive of mania with each individual factor also being significant within the model. In addition to the logistic regression model approach, this hypothesis will also be tested using survival analysis-based Cox regression analyses to predict time to onset of first mania, recognizing that individuals will have variable follow-up times. All analyses will use a backward selection approach to ascertain variables that have unique predictive associations with conversion at an initially liberal threshold of *p* ≤ 0.10. After that, omnibus regression will be conducted in which variables found to contribute uniquely to conversion in the initial series will be considered together. Variables that remain significant at *p* < 0.05 in the omnibus analysis will then be tested for multiplicative (interaction) effects in relation to conversion. Finally, the identified risk factor(s) will be tested regarding their predictive power, calculating sensitivity, specificity, positive predictive value, negative predictive value, and accuracy (e.g., applying ROC analyses). With ≥75 converters (≥15% of *n* = 500 of risk group I), we expect to have >80% power for this model testing. For risk group II, we expect about 4% and for risk group III 5% converters (given a comorbidity rate of about 15%). Formal sample size estimation will be carried out at a pre-planned interim analysis at inclusion of 50% of each risk group into the study. If needed, sample sizes will be adjusted. From the IMAGEN sample, approx. 200 individuals will be included to provide data on the frequency of potential risk factors in this representative population sample.

The outcomes will help to guide future early recognition and intervention by providing a more detailed and reliable approach to psychiatric risk stratification.

### Adjuvant psychotherapy in early stage bipolar disorder (Project A2)

Young bipolar patients (<35 years.) experience the highest personal cost (social, professional, personal) and elevated rates of attempted or completed suicide during the early course of the illness (Perlis et al. 2004). Recent meta-analyses of available clinical trials have concluded that there is limited availability of psychotherapy studies in bipolar disorder patients in general, and no studies exist which have focused on specific subgroups of patients, in particular young adults in the early course of the disorder. However, retrospective data in psychological intervention studies have shown significantly larger effects in patients during early stages of bipolar disorder who had experienced fewer affective episodes (Scott et al. 2007; Meyer and Hautzinger 2012; Reinares et al. 2010). In addition, no intervention study has yet been conducted using neuroimaging methods to identify both predictive factors and neural correlates of successful psychotherapy.

The goal of the proposed randomized controlled trial, which involves 300 younger patients with bipolar disorder in remission, is to test hypothesis that the addition of a specific psychotherapy (SEKT) to psychiatric care (TAU) in comparison to an active (supportive) control treatment (FEST) will lead to positive outcomes such as reduced relapse rates, missed days at work/school, days spent in hospitals, and health costs; significantly improved treatment compliance and social functioning. Participants will receive the option of participating in a neuroimaging study which aims to test the hypothesis that specific psychotherapy (SEKT) will have a more robust effect on neural networks (amygdala, medial prefrontal activation) associated with emotion regulation and social cognition compared to FEST.

#### Intervention and follow-up

Patients between 18 and 35 years of age suffering from bipolar disorder I or II with at least one episode during the preceding 2 years will be eligible for the trial. Participants are required to be in stable remission and in regular medical care, including mood stabilizing medication.

300 patients will be randomized to either SEKT (experimental condition) or FEST (active control condition) and represent the intention-to-treat sample. All included subjects (*n* = 300) will be re-assessed at 6, 12, and 18 months after intake. The recruitment and implementation of therapy will occur at nine clinical sites across the BipoLife consortium.

A new format for patients will be used to deliver both treatment approaches. Over 4 months, 4 full-day workshop-style group treatments with 4–7 patients will be offered by 1–2 specially trained therapists under expert supervision. In the past, this format has proven popular among patients, especially when conducted at weekends, since it is more compatible with occupational or educational schedules.

The SEKT intervention includes elements of cognitive behavior therapy (Lam et al. 2005), interpersonal and social rhythm therapy (Frank et al. 2005), family focused behavior therapy (Miklowitz 2012), mindfulness based therapy (van der Velden et al. 2015), and psychoeducation (Colom et al. 2009). In addition, a new element “emotion and impulsivity regulation skills” will be added. The FEST condition includes general supportive measures and positive feedback, and allows for a more flexible, albeit patient-centered approach.

Relapse will be assessed every 6 months by “Longitudinal follow-up evaluation” (LIFE) (Keller et al. 1987), a structured interview leading to reliable information about course of illness, time in remission, development of new affective episodes (relapse), and comorbidity. In addition, measurements of symptomatology (QIDS-C & QIDS-SR, YMRS, ASRM) (Rush et al. 2003; Young et al. 1978; Altman et al. 1997), social functioning and quality of life (GAF, FAST) (Hall 1995; Rosa et al. 2007) will be assessed. The trial aims to recruit at least 100 participants for the optional neuroimaging study. The paradigms (described in detail in TPP2) will be carried out prior to and following the therapeutic intervention.

The primary efficacy endpoint has been defined as follows: “Relapse” to a new affective (depressive, manic or mixed) episode. This will include a calculation of “relapse rate” and “time to relapse” (days after inclusion to study) assessed by clinician blind to treatment condition using longitudinal follow-up evaluation interview (LIFE) every 6 months. Secondary endpoints will include medication compliance (blood serum level), medication adverse effects, days missed at work/school, and social functioning level. Time until relapse will be addressed by non-parametric survival analyses involving logRank or Wilcoxon statistics and sensitivity analyses for the subgroups ITT and PP. The time-dependent evolution of group-specific hazard rates will be investigated by parametric models addressing differences in the dynamics of relapse in both arms. Cox regressions will be performed for the probability of relapse and time until relapse if the proportional hazards assumption will not be negated. Sample size calculations refer to a logRank statistic under *α* = 0.05 and *β* = 0.2 (80% power) under the assumptions of (a) an accrual time of 24 months, (b) a total study duration of 48 months, and (c) a drop-out rate of 12% (the latter based on experiences from previous studies). We have defined a treatment effect of factor 2 as a relevant effect, i.e., a hazard ratio (HR) of 0.5. The results, if positive, will ideally improve psychotherapeutic content and delivery for young patients in the early stages of bipolar disorder.

### Smartphone-based ambulatory assessment of early warning signs, including personalized real-time data-driven therapeutic interventions, in the long-term treatment of bipolar disorders. A randomized controlled trial (Project A3)

In general, manic and depressive episodes develop during a period of several days to weeks (Strauss et al. 2012). Preventing new episodes is one of the main goals of long-term treatment. To improve early intervention, effective detection of emerging symptoms heralding an imminent episode (early warning signs) is crucial. According to a recent Cochrane review, self-help and psychological treatments that teach patients, alongside other psychological interventions, to recognize and manage early warning signs have been shown to delay time to a new affective episode (Morriss et al. 2007). Since the intervals between visits to the treating psychiatrist are usually long during periods of euthymia, the responsibility to detect early warning signs lies largely with patients themselves. Monitoring affective symptoms, not to miss early warning signs, is typically accomplished with the assistance of mood charting, which, however, has inherent limitations in clinical practice. On the one hand the symptoms of the disorder frequently interfere with patients’ introspective assessment, particularly in the case of emerging mania. On the other hand, the daily documentation becomes tedious during periods of euthymia and is then frequently discontinued.

Smartphones have become a ubiquitous feature of everyday life. In 2015, there were an estimated 46 million smartphone users in Germany, 42 million in the UK, and 2.08 billion users worldwide. (http://www.statista.com/statistics/). Smartphone-based ambulatory assessment (SBAA), including real-time data capture of DSM-5 bipolar disorder symptoms and data-driven, individual threshold-defined therapeutic interventions (SBAA+), represents a promising approach to overcome the limitations of conventional mood charting. The underlying principle is that several parameters of smartphone usage which are likely to be influenced by current psychopathology (GPS, acceleration sensor, communication patterns) are registered automatically via embedded software (Ebner-Priemer et al. 2013), encrypted and transferred to a secure server for real-time analysis. The underlying analyzing algorithm will be informed by previous studies and will utilize the participant’s individual baseline recorded during a period of euthymia as foundation. Patterns breaching the predefined threshold that are compatible with an evolving mood episode can thereby be identified automatically.

The consortium will conduct a randomized, multicenter, observer-blind, parallel group controlled trial with a 78 week (18 month) intervention phase. Five centers across the network will be participating. The primary hypothesis states that continuous ambulatory real-time monitoring of early warning signs for new depressive or (hypo)manic episodes by smartphone-based technology including feedback to the treating physician (SBAA+), will prolong time to a new mood episode and reduce hospitalizations (intervention group). In the control group ambulatory monitoring of early warning signs for emerging depressive or (hypo)manic episodes will occur in an identical manner, but the results will not be transmitted to the treating psychiatrist. The aim is to recruit 180 patients with bipolar disorder for a stabilization phase of up to 6 months. Only those able to maintain euthymia for 4 successive weeks (app. 120) will be randomized to either SBAA+ or SBAA. All patients in this trial, irrespective of their group assignment, will receive guideline-based, state-of-the-art maintenance treatment. The treating psychiatrist will be expected to contact the participant and judge whether an intervention or adaptation of medication is required.

Regarding the primary endpoint, survival analysis (Kaplan–Meier estimators) will be used for estimating time to a new affective episode. Log-Rank tests will be used for group comparisons. All patients who have been randomized will be included in the statistical analyses. For secondary endpoints, two separate survival analyses will be applied to compare the time to a new depressive and (hypo)manic episode, respectively. Wilcoxon-tests for independent samples (SBAA+ vs SBAA) will be used to test for differences in (1) the percentages of assessments meeting the diagnostic criteria, (2) the percentages of assessments hospitalized, (3) the severity of manic symptoms, and (4) the severity of depressive symptoms. The frequencies of adverse events will be compared using Fisher’s exact test. The sample size of *n* = 60 per group was determined from a power calculation for survival analysis with constant hazard and drop-out rates. The relapse rate under SBAA (control condition) was estimated from the patient data from our bipolar disorders outpatient clinic Accordingly, we expect a monthly hazard rate of about 6% which corresponds to a relapse rate of 66% during the observation period of 18 months. From our experience with similar patients from other studies we further expect a monthly drop-out rate of about 2.5% which corresponds to a drop-out rate of 37% during the observation period. The hazard ratio was supposed to be about 0.5.

If shown to be beneficial, ambulatory monitoring may become a further pillar in the maintenance treatment of bipolar disorder for those who choose to use a smartphone (Table 1).

**Table 1:** Methods, study populations, and design within the structure of the BipoLife consortium

Bipolife project	Subject	Population	N	Design	TPP1		TPP2	
					Phenotype database	Biomaterials	Neuroimaging	EEG
A1	Assessment of factors associated with conversion to bipolar disorder	Young persons at-risk of BD	1500	Epidemiological, Follow-up	✓	✓	✓	✓
A2	Trial of specific group psychotherapy for bipolar disorder	Young patients with recent onset of BD	300	Double-blind, randomized, controlled trial	✓	✓	✓	
A3	Smartphone-based ambulatory monitoring with threshold based feedback	Patients with a highly recurrent/unstable course of BD	180	Rater blind, randomized, parallel group trial	✓			
B2	Neuroimaging study in patients with affective disorders and acute suicidality	Patients with severe suicidality	80	Double-blind, randomized, placebo-controlled trial	✓		✓	
B3	Genomic, transcriptomic and proteomic markers of lithium response	BD patients with lithium response vs. non response	150	Cross-sectional	✓	✓		

### Neuroimaging markers for the prediction of lithium treatment effects in a suicidal depressive episode of bipolar disorder (Project B2)

Lithium is an established substance for the prevention of suicide in affective disorders (Lewitzka et al. 2015b). It remains uncertain, however, whether these effects can also be observed in the short-term treatment of acutely suicidal patients. Within a separate multicenter, double-blind, randomized controlled trial investigating the effect of lithium in addition to TAU in affectively ill patients with acute suicidality over 5 weeks (Lewitzka et al. 2015a), a neuroimaging study will aim to describe the neural correlates of lithium treatment on suicidality. Furthermore, the project aims to identify neuroimaging markers that are associated with and may be predictive of treatment response with lithium. Participants will receive an fMRI prior to and following the 5 week intervention. The neuroimaging study will be carried out at five sites within the consortium and aims to recruit a total of 80 participants. If successful, neuroimaging data may in future assist in more rational therapeutic decision-making.

### Genomic and transcriptomic biomarkers for predicting lithium treatment response in bipolar disorder (Project B3)

Lithium remains the mainstay of treatment in bipolar disorder (Nolen 2015; Severus et al. 2014). Although a substantial number of patients respond excellently to lithium monotherapy, this is not true for all (Kessing et al. 2011). Currently, there are no reliable biomarkers which could predict lithium response, leaving physician and patient to establish the suitable treatment by trial and error. This project aims to delineate a “lithium predictor tool set” integrating information from genome-wide association study (GWAS) data and transcriptomic analyses.

Using the already available unique phenotype and biobank resource of the Consortium on Lithium Genetics (http://www.ConLiGen.org), lymphoblastoid cell lines (LCLs) from 75 lithium responders (R) and 75 non-responders (R) BD patients will be investigated by the use of genome-wide expression profiling of genes and miRNAs. Applying a replication design, an exploratory analysis comparing LCLs from 30 R and 30 NR patients will be performed first. Best results will be followed up in the remaining 45 R and 45 NR individuals. Analyses will be performed using real-time PCR for the top genes and miRNAs from the exploratory part. We will apply a cutoff value of a 1.5-fold difference between the groups at *p* < 0.001. Cellular drug effects will be studied, using lithium-mediated growth inhibition that will be correlated to clinical response and gene expression profiling in LCLs from the patient sample characterized for lithium response and control individuals. Using state-of-the art bioinformatics approaches, the project will be the first to combine information obtained from GWAS and expression profiling to define a set of genomic predictors of lithium response. This may develop to become a future tool for a more personalized treatment approach to mood stabilization in BD.

### Neuroimaging, neurophysiology, biobanking, and data-management infrastructure (Projects TPP1 and TPP2)

Within a research network of this size conventional methods of data acquisition (i.e., paper and pencil), storage (i.e., local hard drive or server), and processing (i.e., spreadsheet) would be inadequate, slow, and unsafe. In addition, the requirements of funding bodies, ethics committees, and regulatory authorities are becoming ever more rigorous. These challenging demands will be met by a centralized framework for highly encrypted proband and identity management; flexible and swift storage, retrieval and exchange of data, and biomaterial. Data acquisition will occur at all sites via a browser-based interface using SecuTrial (http://www.secutrial.com) software. The data storage will be managed by the medical informatics section of Göttingen University. Biomaterials will be acquired using consortium-wide SOPs and protocols and all 2D barcoded material will be stored at two mirrored sites in Gottingen and Würzburg for genomic, transcriptomic, and proteomic analyses.

Acquiring neuroimaging data from nine different sites requires the harmonization of protocol, standardization of paradigms, and regular quality control to detect and combat local signal-aberrations early (Hellerbach et al. 2013). All participants opting for the additional neuroimaging study within A1, A2 or B2 will receive an identical battery including:

i. T1-sequence for morphometric analyses
ii. T2-sequence for investigating the hippocampus on a subregional level
iii. Resting-state fMRI sequence and three fMRI activation paradigms
iv. Highly robust emotional face matching paradigm for assessing limbic responsiveness to negative facial expressions (Dannlowski et al. 2012).
v. Cartoon Theory-of-Mind task which robustly activates the theory of mind (ToM) network relevant for social cognition, since a dysfunction of the ToM network activated by this task is associated with a genetic risk variant for BD and in relatives of patients with BD (Walter et al. 2011).
vi. Desire-reason dilemma task using conditioned reward stimuli in different experimental situations, thereby allowing the investigation of subcortical structures of the dopaminergic reward system and their specific functional interactions with prefrontal cortices (Diekhof and Gruber 2010).

An EEG battery focusing on neural synchrony in long-range and local oscillatory responses will include cognitive (choice-reaction tasks), perceptive (Kanizsa figures), and emotional (emotional faces) paradigms (Özerdem et al. 2011), complementing the MRI data.

## Conclusions

The German BipoLife consortium consists of a nationwide network of university centers aiming to address unanswered questions in the early recognition, reliable diagnosis, rational treatment, and prognosis of bipolar disorder. By utilizing a centralized secure IT-infrastructure, the consortium aims to minimize incomplete data and provide safe and rapid access for analyses (i.e., interim analysis). The consortium hopes to establish fluid and neuroimaging biomarkers to improve rational treatment decisions and support the estimation of prognosis. The studies will hopefully also encourage more effective ways of early warning sign detection and more convenient delivery of psychotherapeutic care.

The structure of the gathered data will deliver the opportunity for complex secondary analyses, combining highly detailed phenotypic, neuroimaging, genomic, and transcriptomic information.

The crucial aim within the BipoLife consortium is to lay the foundation for a contemporary research infrastructure specific for bipolar disorder and the network partners will campaign to secure a more permanent funding.
